# Flow-Injection Coulometric Detection Based on Ion Transfer and Its Application to the Determination of Chlorpromazine

**DOI:** 10.3390/s8063678

**Published:** 2008-06-01

**Authors:** Joaquín A. Ortuño, Antonio Gil, Concepción Sánchez-Pedreño

**Affiliations:** Department of Analytical Chemistry, Faculty of Chemistry, Campus de Espinardo, University of Murcia, 30071-Murcia, Spain

**Keywords:** Interface between Two Immiscible Electrolyte Solutions, ion transfer, coulometry, chlorpromazine determination, flow-injection analysis

## Abstract

A flow-injection coulometric method for the determination of chlorpromazine based on ion transfer into a plasticized poly(vinyl chloride) (PVC) membrane, was developed. The detector used consists of a flow-through cell that incorporates a plasticized poly(vinyl chloride) (PVC) membrane which contains tetrabutylammonium tetraphenylborate as electrolyte. The membrane is located between the flowing solution and an inner aqueous electrolyte solution. Two pairs of electrodes, each pair formed by a reference electrode and a working electrode, are used, one pair in each solution. The potential between the reference electrodes was controlled by a four-electrode potentiostat with ohmic drop compensation. A potential step capable of producing the transfer of the chlorpromazine ion into the membrane was applied during the passage of a wide portion of sample plug through the cell and the corresponding quantity of the electricity was measured. In the selected conditions, a linear relationship was observed between the quantity of electricity and chlorpromazine concentrations over a range of 1×10^-6^ −1×10^-4^ M. The detection limit was 2 × 10^-7^ M. Good repeatability and between-day reproducibility was obtained. No interference was observed on the part of some common ions and pharmaceutical excipients. The method proposed was applied satisfactorily to the determination of chlorpromazine in pharmaceuticals and human urine.

## 1. Introduction

Ion transfer across the Interface between Two Immiscible Electrolyte Solutions (ITIES) is a fundamental aspect of the Electrochemistry of Liquid-Liquid Interfaces and several reviews [1-3] deal with its fundamentals, methodology and applications. The use of ion transfer across ITIES to develop detection systems for flow-injection analysis is limited by the mechanical instability of the liquid/liquid interfaces. However, several flow-injection amperometric methods for the determination of different ions based on ion transfer across ITIES has been reported [4-13]. In these methods, the liquid/liquid interface is stabilised in two main ways, using a dialysis membrane located between both phases [4-6] or increasing the viscosity of the organic phase with poly(vinyl chloride) (PVC) to form a polymer gel [7-8] which can be arranged as an array of micro-interfaces [9], or even a plasticized PVC membrane [10-13] similar to those used in ion-selective electrodes (ISEs). Due to their physical and chemical properties [14], the membranes of the last type can be easily accommodated in flow-through cells, which they exhibit a long lifetime. Differential pulse voltammetric techniques have been introduced to accurately determine the standard ion transfer potentials into these membranes [15-16], which govern the sensitivity and selectivity of the corresponding electrochemical sensors. Interestingly, a relationship between the values found for the transfer of some ionic drugs and their corresponding pharmacological potency was found [16].

The main advantages of using amperometric detection methods based on ion transfer at ITIES over classical potentiometric detection using ion-selective electrodes are that the ion selectivity of the former can be tuned by altering the magnitude of the applied potential and that the amperometric response is directly proportional to analyte concentration, which is more suitable for detecting small changes in analyte concentration than the more usual potentiometric ISEs, which provide a potential response proportional to the logarithm of the analyte activity.

In the present paper, a flow-injection coulometric method for the determination of chlorpromazine is proposed, in which the quantity of electricity corresponding to the chlorpromazine ion transfer into a plasticized polymeric membrane during the passage of the central portion of the sample plug through the detector cell is measured. Some principles of this technique have previously been presented for the transfer of the model ion tetraethylammonium [17]. Some authors have used the coulometric detection based on ion transfer in flow systems [18-19].

Chlorpromazine is derived from phenothiazine and has an aliphatic side chain typical of low to middle potency neuroleptics. Chlorpromazine has antipsychotic and antiemetic effects, its generic name being chlorpromazine hydrochloride, (2-Chloro-10-[3-dimethylamino-propyl] phenothiazine hydrochloride (Scheme 1).

Several methods for the determination of chlorpromazine using different techniques, including spectrophotometry [20], spectrofluorimetry [21], chemiluminescece [22], spectroelectrochemistry [23], polarography [24], voltammetry [25], potentiometry with an ion-selective electrode [26-27], liquid chromatography [28-29] and capillary electrophoresis [30], have been reported. The coulometric method here proposed is applied to the determination of chlorpromazine in pharmaceuticals and human urine.

## 2. Experimental Section

### 2.1 Apparatus

The home-built four-electrode potentiostat and four-electrode flow cell used were described in [17]. Ag/AgCl electrodes were used as reference electrodes and Pt electrodes were used as counter electrodes. A one-channel flow-injection assembly was used. The distance between the injection valve and the cell was 30 cm. A Merck Hitachi L-7110 isocratic pump, Omnifit injection valve, PTFE tubing of 0.5 mm bore and various end fittings and connectors were used to construct the flow injection system. A glass ring of 28 mm inner diameter and 30 mm height, glass plate, vial and punch were purchased from Fluka for the construction of the membranes.

### 2.2 Reagents and solutions

Poly(vinyl chloride) (PVC) high molecular mass, 2-nitrophenyl octyl ether (NPOE), and tetrahydrofuran (THF) were Selectophore products from Fluka. Tetrabutylammonium tetraphenylborate (TBATPB), tetrabutylammonium chloride (TBACl), (N-[2-Hydroxyethyl] piperazine-N′- [2-ethanesulfonic acid] sodium salt (HEPES) and chlorpromazine hydrochloride (CzHCl) were purchased from Sigma. A 1×10^-2^ M solution of CzHCl was prepared by dissolving in water. Working solutions were prepared by diluting with 5×10^-2^ M LiCl. Glucose, lactose, sucrose and starch were purchased from Probus. All the other reagents used were of analytical reagent grade. Nanopure water (18-M Ω) prepared with a Milli-Q (Millipore) system was used throughout.

Largactil tablets (Laboratoires Aventis, Paris, France): 25 mg chlorpromazine hydrochloride, starch, lactose, sucrose and other excipients. Largactil ampoule (Laboratoires Aventis, Paris, France): 25 mg chlorpromazine hydrochloride by ampoule, and other excipients.

### 2.3 Membrane preparation

The membranes were prepared by dissolving 200 mg NPOE, 100 mg PVC and 8.4 mg TBATPB in 3 mL of tetrahydrofuran. This solution was poured into the glass ring resting on the glass plate and was left overnight to allow the solvent to evaporate slowly. A 7-mm diameter piece was cut out with the punch and incorporated into the flow-through cell. The electrochemical cell can be expressed as

$$\underset{↔}{\text{Ag}\left|\text{AgCl}\right|5\times 10^{‐2}\text{M TBACl}\left|5\times 10^{‐2}\text{M TBATPB}\right|}\underset{↔}{5\times 10^{‐2}\text{M LiCl},\text{xM CzHCl}\left|\text{AgCl}\right|\text{Ag}}$$

The applied potential *E* is defined as the potential difference between the right and left hand terminals. *E* is controlled by means of the four-electrode potentiostat that applies the necessary potential between the right and left counter electrodes. A positive current corresponds to the transfer of cations from the sample phase into the membrane phase.

### 2.4 Flow-injection coulometric procedure for the determination of chlorpromazine

Aliquots of 160 ml of chlorpromazine working solutions (1×10^-6^ − 1×10^-4^ M) were injected in a 5×10^-2^ M LiCl carrier solution pumped at a flow rate of 1.0 ml min^-1^, while applying a double potential step. This consisted of an initial potential of 0.250 V for 8 s, after which the potential was stepped to a forward potential of 0.375 V for 10 s, then to 0.200 V for 50 s. The current versus time response curve was recorded and the corresponding quantity of electricity versus time was obtained by integration. The quantity of electricity obtained during the application of the first potential step (0.375 V) was plotted versus CzHCl concentration. A blank assay was also run to correct the values.

### 2.5 Flow-injection coulometric procedure for the determination of chlorpromazine in pharmaceuticals and human urine

Tablets: The content of ten tablets was mixed and weighed. An amount of 3.7 mg of tablet powder was accurately weighed and dissolved in 50 mL of 5×10^-2^ M LiCl, 5×10^-2^ M HEPES pH 7.4. The chlorpromazine concentration was determined following the procedure described in *2.4* but using a calibration graph realised in the same medium as the sample.

Ampoule: The content of three ampoules was transferred to a 500-mL calibrated flask and diluted to the mark with 5×10^-2^ M LiCl, 5×10^-2^ M HEPES pH 7.4 solution. The chlorpromazine concentration was determined as described above.

Human urine: Samples were obtained from a healthy volunteer. In the absence of urine samples containing chlorpromazine, known amounts of chlorpromazine hydrochloride were added. The chlorpromazine was extracted following a procedure similar to that described in [24]. The pH of a 50 mL sample of urine was adjusted to 10 with 1.0 M NaOH and 5 mL n-hexane were added. The solution was shaken and the aqueous phase was discarded. The organic phase was shaken with 5 mL 5×10^-1^ M H_2_SO_4_ solution and the aqueous phase was separated, collected into a 10-mL calibrated flask and diluted to the mark with 1×10^-1^ M LiCl. The chlorpromazine concentration was determined as described in *2.4*. A urine sample containing no chlorpromazine was also run as a blank.

## 3. Results and Discussion

**Figure 1.**
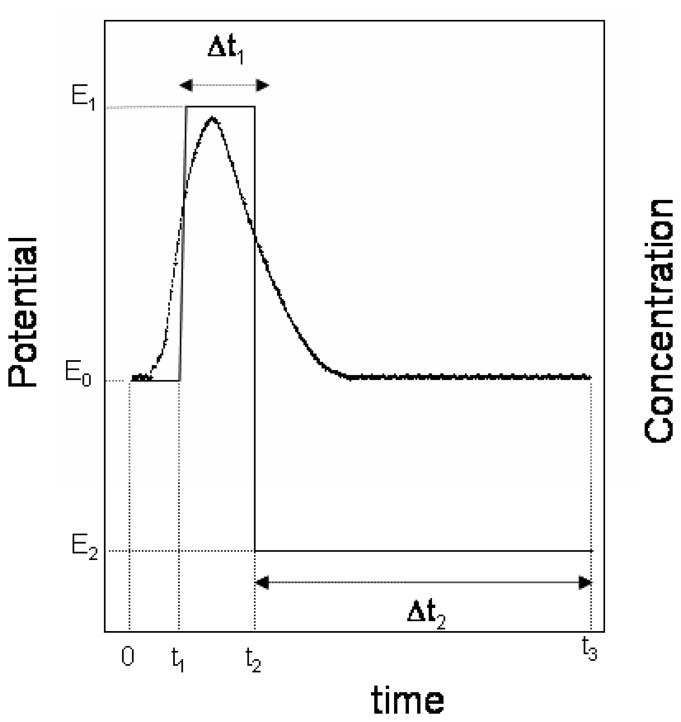
Principle of the method. Applied potential (left) and concentration of the sample plug (right) vs. time. The concentration profile was obtained from the amperometric response of the detector after injecting a sample, applying a constant potential of 0.375 mV to the detector.

**Figure 2.**
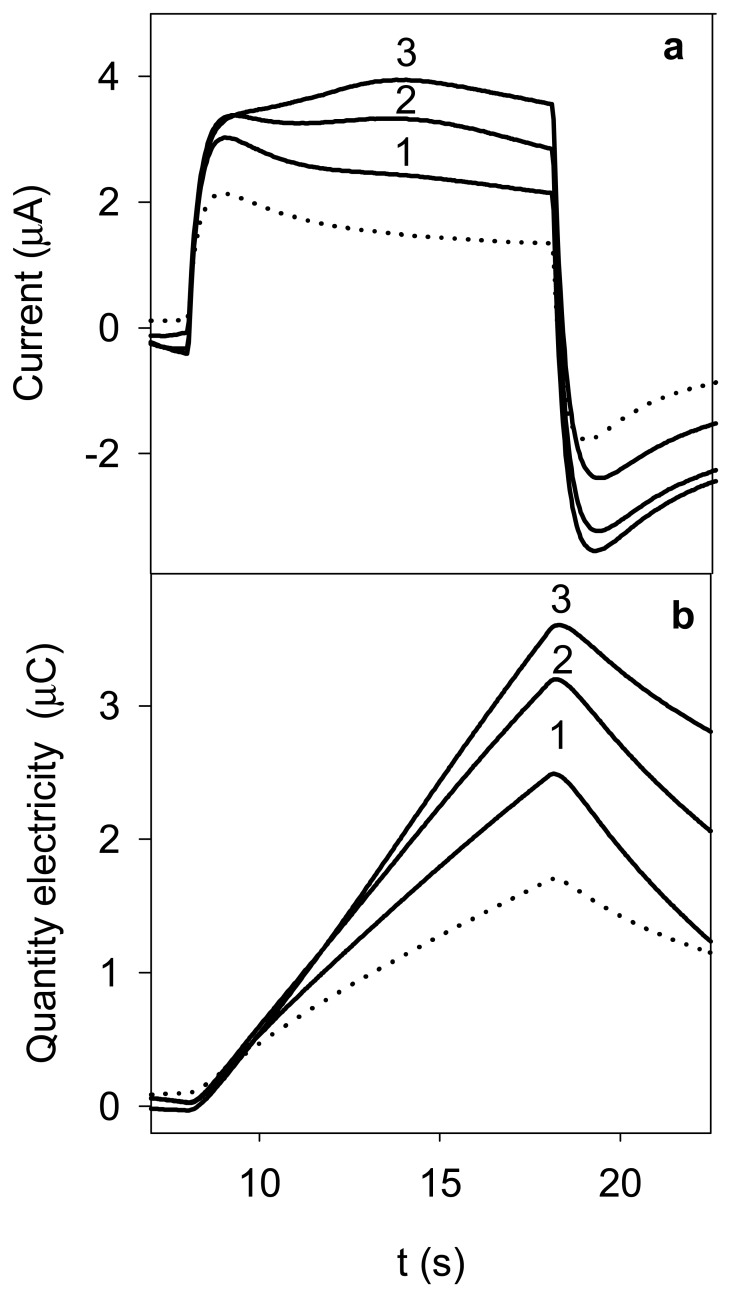
Current (a) and quantity of electricity (b) vs. time curves obtained for: (1) 4 × 10^-5^ M, (2) 8 × 10^-5^ M, (3) 1 × 10^-4^ M chlorpromazine and (•••) blank. E_1_ = 0,375 V.

The principle of the flow-injection coulometric method is shown in Fig.1, and the experimental results obtained following the procedure described in *2.4* for three chlorpromazine concentrations are shown in Fig.2. As shown in Fig.1, a double potential step was applied at the same time as the sample was injected. As can be seen in Fig.2, at the initial potential applied (*E*_0_), the background current corresponding to the supporting electrolyte was negligible. At time *t*_1_ (Fig.1), when the central portion of the sample plug had arrived in the detector cell, a forward potential *E*_1_ capable of causing the transfer of protonated chlorpromazine cation into the membrane was applied for a duration Δ*t_1_* = *t_2_* − *t_1_*, which produced a positive transient current response (Fig. 2a). The quantity of electricity sampled at time *t*_2_ (Fig.1) corresponds to the peak value in Fig. 2b. As can be seen this peak value increased as the chlorpromazine concentration in the injected sample increased. The potential was then stepped to the potential *E*_2_ (Fig.1), at which the chlorpromazine cation in the membrane was transferred to the flowing solution, producing a negative transient current response (Fig.2a). The corresponding process that takes place at the inner solution/membrane interface is the transfer of tetrabutylammonium from the membrane to the internal solution and vice versa, this behaving as an ideally non-polarizable interface because of the presence of a high concentration of tetrabutylammonium in both sides. Before a new injection, the potential was stepped to the initial value *E*_0_, which was held sufficient time to reach a zero current value.

With regard to the origin of the current observed for the blank, we think that it is mainly a faradic current arising from the transfer of ions of the supporting electrolytes. Taking into account the standard transfer potential values reported for lithium and tetraphenylborate ions between water and a polyvinyl chloride membrane plasticized with 2-nitrophenyl octyl ether [31], the blank current would be caused by the egress of tetraphenylborate from the membrane.

### 3.1 Influence of the electrochemical variables

The t_1_ value was tested to synchronize it with the passage of the more concentrated portion of the sample plug through the detector cell. A value of 8 s was found and used in further studies.

**Figure 3.**
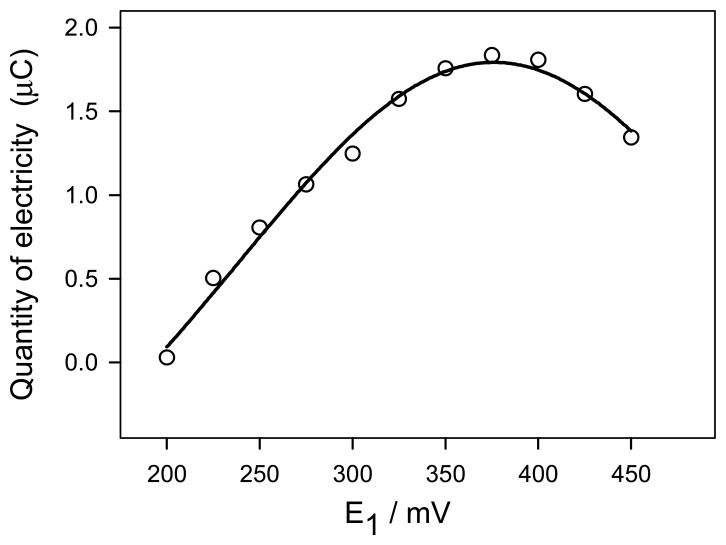
Influence of the applied potential E_1_.

The influence of the applied potential *E*_1_ on the quantity of electricity corresponding to chlorpromazine transfer was studied in the range 200-500 mV using a 1.0 × 10^-4^ M CzHCl working solution and the remaining experimental conditions described in *2.4*. The results are shown in Fig. 3. As can be seen, a maximum quantity of electricity was obtained at a potential E_1_ of 0.375 V and this value was selected for further studies.

The influence of the time interval Δt_1_ was studied in the range 2-20 s. The blank values and the quantity of electricity corresponding to chlorpromazine transfer increased as the time Δt_1_ was increased over the whole range studied. Also, the reproducibility was worse at the highest time values assayed. A possible explanation of the above is that for these Δt_1_ values, a Δt_2_ of 50 s is not sufficient for the complete regeneration of the membrane. Taking all this in to account a Δt_1_ value of 10 s was selected for further studies.

### 3.2 Influence of pH

**Figure 4.**
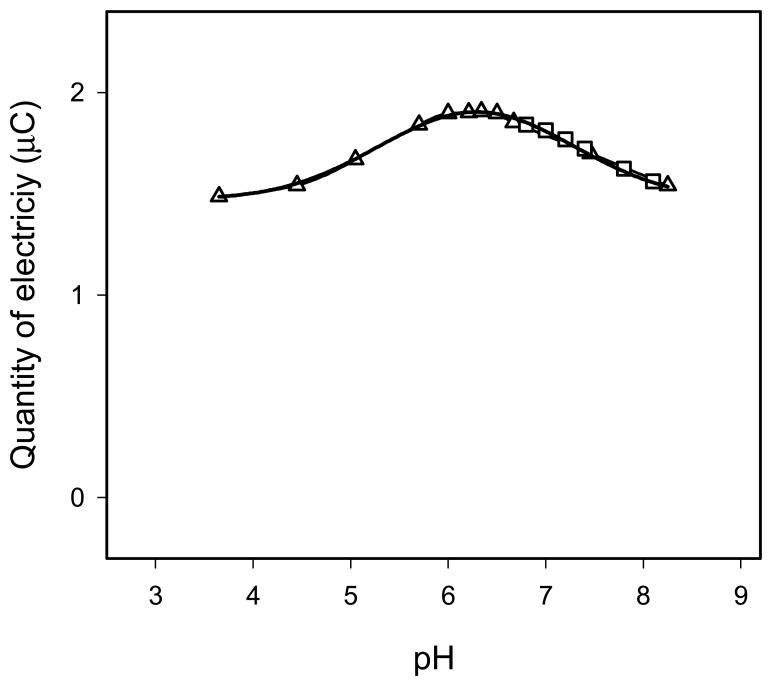
Influence of pH. (Δ) pH adjusted with H_2_SO_4_ or NaOH. □ pH adjusted with HEPES buffer.

The influence of the pH was studied over the range 3.7-8.3 by adding H2SO4 or NaOH solutions, in the selected conditions described in *2.4* using a 1.0 × 10^-4^ M CzHCl working solution. The influence of pH was also studied over the range 6.8-8.1 by adjusting the pH with HEPES buffer. The results obtained are shown in Fig.4. As can be seen, the plot of the quantity of electricity versus pH had a maximum at about pH 6.0. There was no difference between the results obtained by adjusting the pH values with H_2_SO_4_/NaOH or HEPES buffer. The pH of the chlorpromazine working solutions prepared in 5 × 10^-2^ M LiCl was 5.7, which lies close to the maximum.

### 3.3 Features of the method

The calibration graph for chlorpromazine was studied between 1×10^-6^ and 1×10^-3^- M (0.36-355 μg mL- chlorpromazine hydrochloride) following the procedure in *2.4*. A linear relationship between the peak height (H) and imipramine concentration was obtained up to 1×10^-4^ M CzHCl. The regression equation was H(μC) = 1.8×10^4^ [CzHCl] (M) + 2.3×10^-3^ with a regression coefficient of 0.998. The detection limit obtained from three times the standard deviation of the baseline noise was 2.0 × 10^-7^ M.

The repeatability of the proposed method was evaluated by performing ten consecutive injections of 2×10^-5^ M and 8×10^-5^ M CzHCl working solution. The variation coefficients obtained were ±1.3 and ±1.5%, respectively. The between-day reproducibility was studied by injecting a 5×10^-5^ M CzHCl working solution on ten random days during a two week period. During this period, the detector cell was stored with the supporting electrolyte solutions inside. The variation coefficients of the means corresponding to three injections each day were ±1.6 %.

A comparison of the sensitivity of the proposed method with those corresponding to the other methods referred to for the determination of chlorpromazine [20-30] shows that the proposed method is more sensitive than the spectrophotometric, spectroelectrochemical and potentiometric methods, similar in sensitivity to the voltammetric, spectrofluorimetric, polarographic and solid phase extraction-liquid chromatographic methods and less sensitive than direct HPLC and electrophoretic methods. An advantage of the proposed method over former methods is its lower cost and greater simplicity.

### 3.4 Effect of other species

The effect of other species on the coulometric method for the determination of chlorpromazine was studied by injecting solutions of 5×10^-5^ M CzHCl working solution containing different concentrations of several inorganic and organic species. Cation solutions were in the form of sulphates and nitrates, and anion solutions in the form of sodium salts. The tolerance limit was taken as the concentration causing an error of not more than ±5 % in the determination of chlorpromazine. K^+^, Na^+^, Ca^2+^, Mg^2+^, Cl^-^, glucose, lactose, sucrose and starch did not interfere, at least up to a species/chlorpromazine molar ratio of 100. In view of the results found in previous papers on amperometric methods based on ion transfer across a water-plasticized polymeric membrane interface [11-12], the interference of the pharmaceutical compounds imipramine and verapamil is expected.

### 3.5 Applications

The flow injection coulometric method proposed was applied to the determination of chlorpromazine hydrochloride in pharmaceuticals and human urine following the procedures described in *2.5*. The results obtained are shown in Table 1, where they are compared with the labelled or added values. Excellent recoveries were obtained.

### 3.6 Conclusions

The flow-injection coulometric method based on ion transfer into a plasticized poly(vinyl chloride) (PVC) membrane permits the determination of chlorpromazine with good sensitivity and reproducibility. The method proposed can be used for the determination of chlorpromazine hydrochloride in pharmaceuticals and human urine.

## Figures and Tables

**Figure:**
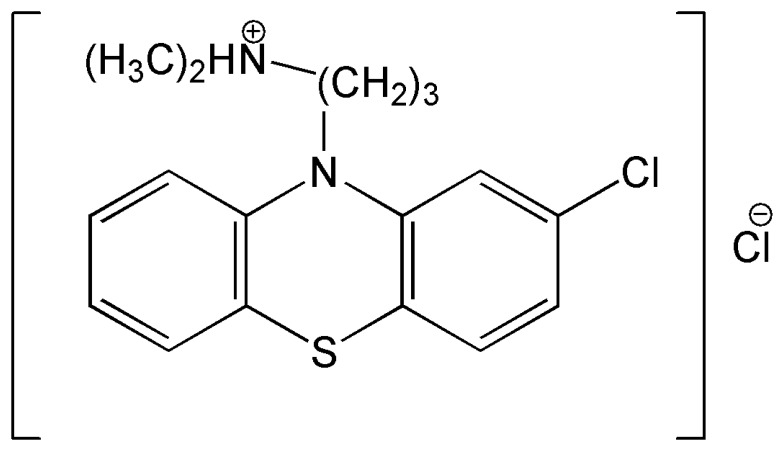
Scheme 1.

**Table 1.** Determination of chlorpromazine hydrochloride in pharmaceuticals and urine.

Sample	Labelled or added	Found^a^	Recovery (%)
Largactil tablet	25^b^	24.6 ± 0.4	98.6
Largactil ampoule	25^b^	25.1 ± 0.2	100.6
Urine	0.89^c^	0.87 ± 0.02	97.8
	7.11^c^	7.29 ± 0.07	102.5
	17.76^c^	18.25 ± 0.04	102.8
	28.40^c^	29.30 ± 0.42	103.2

a Mean value ± standard deviation (three determinations)

b mg chlorpromazine hydrochloride / ampoule or tablet

c μg mL^-1^ chlorpromazine hydrochloride

## References

[1] Reymond F., Fermín D., Lee H.J, Girault H.H. (2000). Electrochemistry at liquid/liquid interfaces: methodology and potential applications”. Electrochim. Acta.

[2] Samec Z., Samcová E., Girault H.H. (2004). Ion amperometry at the interface between two immiscible electrolyte solutions in view of realizing the amperometric ion-selective electrode. Talanta.

[3] Samec Z. (2004). Electrochemistry at the interface between two immiscible electrolyte solutions. Pure Appl. Chem..

[4] Wilke S., Franzke H., Müller H. (1992). Simultaneous determination of nitrate and chloride by means of flow-injection amperometry at the membrane-stabilized water/nitrobenzene interface. Anal. Chim. Acta.

[5] Sawada S., Torri H., Osakai T., Kimoto T. (1998). Pulse Amperometric Detection of Lithium in Artificial Serum Using a Flow Injection System with a Liquid/Liquid-Type Ion-Selective Electrode. Anal. Chem..

[6] Dunaeva A.A., Wilke S., Kolycheva N.V., Petrukhin O.M., Muller H. (1999). Determination of alkali metal ions by flow-injection amperometry at the interface of two immiscible electrolyte solutions in the presence of dicyclohexyl-18-crown-6. J. Anal. Chem..

[7] Marecek V., Janchenova H., Colombini M.P., Papoff P. (1987). Charge transfer across a polymer gel/liquid interface: A voltammetric detector for a flow system. J. Electroanal. Chem..

[8] Ji H., Wang E. (1988). Flow injection amperometric detection based on ion transfer across a water-solidified nitrobenzene interface for the determination of tetracycline and terramycin. Analyst.

[9] Lee H.J., Pereira C.M., Silva A.F., Girault H.H. (2000). Pulse Amperometric Detection of Salt Concentrations by Flow Injection Analysis Using Ionodes. Anal. Chem..

[10] Ortuño J.A., Hernández J., Sánchez-Pedreño C. (2004). Flow-injection amperometric detection with solvent polymeric membrane ion sensors. Electroanal.

[11] Ortuño J.A., Sánchez-Pedreño C., Gil A. (2005). Flow-injection pulse amperometric detection based on ion transfer across a water-plasticized polymeric membrane interface for the determination of verapamil. Anal. Chim. Acta.

[12] Ortuño J.A., Sánchez-Pedreño C., Gil A. (2007). Flow-injection pulse amperometric detection based on ion transfer across a water-plasticized polymeric membrane interface for the determination of imipramine. Sens. Actuators B: Chemical.

[13] Ortuño J.A., Rueda C. (2007). Flow-injection amperometric determination of tacrine based on ion transfer across a water-plasticized polymeric membrane interface. Sensors.

[14] Armstrong R.D., Horvai G. (1990). Properties of PVC based membranes used in ion-selective electrodes. Electrochim. Acta.

[15] Ortuño J.A., Serna C., Molina A., Gil A. (2006). Differential pulse voltammetry and additive differential pulse voltammetry with solvent polymeric membrane ion sensors. Anal. Chem..

[16] Ortuño J.A., Gil A., Serna C., Molina A. (2007). Voltammetry of some catamphiphilic drugs with solvent polymeric membrana ion sensors. J. Electroanal. Chem..

[17] Sánchez-Pedreño C., Ortuño J.A., Hernández J. (2002). Chronocoulometric flow-injection analysis with solvent polymeric membrane ion sensors. Anal. Chim. Acta.

[18] Sawada S., Taguma M., Kimoto T., Hotta H., Osakai T. (2002). Complete electrolysis using a microflow cell with an oil/water interface. Anal. Chem..

[19] Yoshizumi A., Uehara A., Kasuno M., Kitatsuji Y., Yoshida Z., Kihara S. (2005). Rapid and coulometric electrolysis for ion transfer at the aqueous|organic solution interface. J. Electroanal. Chem..

[20] Eldin F., Suliman O., Sultan S.M. (1994). Sequential optimization of a flow injection spectrophotometric method for the assay of chlorpromazine in pharmaceutical preparations. Talanta.

[21] Fardous A.M., Horria A.M., Samiha A., Sameh A.A. (2005). A validated spectrofluorimetric method for determination of some psychoactive drugs. J. Pharm. Biomed. Anal..

[22] Pérez-Ruiz T., Martínez Lozano C., Sanz A., San Miguel M.T. (1999). Flow-injection chemiluminescent determination of phenothiazines in pharmaceutical preparations. Lab. Autom. Inf. Manage..

[23] Daniel D., Gutz I.G.R. (2005). Spectroelectrochemical determination of chlorpromazine hydrochloride by flow-injection analysis. J. Pharm. Biomed. Anal..

[24] Belal F., El-Ashry S.M., Shehata I.M., El-Sherbeny M.A., El-Sherbeny D.T. (2000). Differential-pulse polarographic determination of some N-substituted phenothiazine derivates in dosage forms and urine through treatment with nitrous acid. Mikrochim. Acta.

[25] Ni Y., Wang L., Kokot S. (2001). Voltammetric determination of chlorpromazine hydrochloride and prometazine hydrochloride with the use of multivariate calibration. Anal. Chim. Acta.

[26] Sales M.G.F., Tomas J.F.C., Lavandeira S.R. (2006). Flow injection potentiometric determination of chlorpromazine. J. Pharm. Biomed. Anal..

[27] Ortuño J.A., Sánchez-Pedreño C., Hernández J. (2006). Ion-selective electrode for the determination of some multidrug resistance reversers. Sens. Actuators B: Chemical.

[28] Pistos C., Stewart J.T. (2003). Direct injection HPLC method for the determination of selected phenothiazines in plasma using a Hisep column. Biomed. Chromatogr..

[29] Quintana M.C., Ramos L., González M.J., Blanco M.H., Hernández L. (2004). Development of a solid phase extraction method for simultaneous determination of corticoids and tranquilizers in serum simples. J. Sep. Sci..

[30] Lara F.J., García-Campaña A.M., Alés-Barrero F., Bosque Sendra J.M. (2005). Development and validation of a capillary electrophoresis method for the determination of phenothiazines in human urine in the low nanogram per milliliter concentration range using field amplified sample injection. Electrophoresis.

[31] Langmaier J., Stejskalová K., Samec Z. (2001). Evaluation of the standard ion transfer potentials for PVC plasticized membranes from voltammetric measurements. J. Electroanal. Chem..

